# The application of graph theory and percolation analysis for assessing change in the spatial configuration of pond networks

**DOI:** 10.1007/s11252-017-0724-8

**Published:** 2017-12-18

**Authors:** Ian Thornhill, Lesley Batty, Matthew Hewitt, Nikolai R. Friberg, Mark E. Ledger

**Affiliations:** 1 School of Geography, Earth and Environmental Sciences, University of Birmingham, Edgbaston, Birmingham, West Midlands B15 2TT UK; 2 CoLA - Culture and Environment, Bath Spa University, Newton Saint Loe, Bath, BA2 9BN UK; 3 Norwegian Institute for Water Research (NIVA), Gaustadalléen 21, 0349 Oslo, Norway; 4 water@leeds, School of Geography, University of Leeds, Leeds, LS2 9JT UK

**Keywords:** Ponds, Fragmentation, Stepping stones, Resilience, Minimum spanning tree, Urban ecology

## Abstract

Pond networks support high levels of biodiversity when compared to other freshwater ecosystems such as rivers, lakes and streams. The persistence of species in these small, sometimes ephemeral, aquatic habitats depends on the dispersal of individuals among ponds in the landscape. However, the number of ponds across the landscape is at a historical low as urbanisation and intensified agricultural practices have led to a substantial loss of ponds (nodes in the pond network) over more than a century. Here, we examine the extent and drivers of pond loss in a heavily urbanised landscape (Birmingham, UK) over 105 years and determine how pond loss influences key structural properties of the pond network using graph theoretic approaches. Specifically, we calculated minimum spanning trees (MST) and performed percolation analyses to determine changes in both the spatial configuration and resilience of the pond network through time. Pond numbers declined by 82% between *ca*1904 and 2009, such that pond density decreased from 7.1 km^-2^ to 1.3 km^-2^. The MST analyses revealed increased distance between ponds in the network (i.e. edge length increased) by up to 49% over the 105-year period, indicating that ponds in the modern landscape (2009) were considerably more isolated, with fewer neighbours. This study demonstrates that graph theory has an excellent potential to inform the management of pond networks in order to support ecological communities that are less vulnerable to environmental change.

**Electronic supplementary material:**

The online version of this article (10.1007/s11252-017-0724-8) contains supplementary material, which is available to authorized users.

## Introduction

Ponds are discrete aquatic habitats distributed across the terrestrial landscape to form a naturally fragmented network, or 'pondscape' (Boothby 1999) and many pond-dwelling organisms are effective dispersers that have the capacity to move long distances between pond habitats in order to acquire resources, avoid predators, competitors, and disturbance, and seek out conspecifics (Fahrig 2007). As a consequence, local populations in ponds become linked by the movement of individuals to form metapopulations sustained across the wider pond network (e.g. Jeffries 1994; Briers et al. 2004). Therefore, the ability of pond-dwelling organisms to disperse among ponds is especially pivotal in promoting species persistence in a dynamic habitat network (Gibbs 2000; Fortuna et al. 2006) in which individual ponds are gained and/or lost through time via a range of natural and anthropogenic processes (Jeffries 2012).

Land-use change such as urbanisation can limit the natural processes that create ponds such as erosional processes or floodplain dynamics (Indermuehle et al. 2008; Williams et al. 1998b, 2010) and accelerate the destruction of natural ponds (Sukopp 1981) or those used formerly for agriculture or industrial purposes (Wood and Barker 2000). This loss and destruction of pond habitat is common to many countries across the world (Fairchild et al. 2013; Hassall 2014). Across the United Kingdom (UK), 32% of ponds are estimated to have been lost over 120 years between 1880 and 2000: a rate of 0.27% per year (Biggs et al. 2005). Losses have occurred in both rural and urban areas, however, the greatest loss (>80%) has been estimated for urban areas such as London between 1870 and 1984 (Langton 1985) and the city of Cardiff (Rich 1998) or areas of intensive agriculture (Beresford and Wade 1982). These major declines are likely to mask a relatively high turnover of sites as ponds are lost and gained over time (Williams et al. 1998a). Some evidence has emerged to suggest that pond losses may have slowed or reversed recently (Biggs et al. 2005; Williams et al. 2010), potentially as pond creation has become imbedded within amenity developments (Jeffries 2012), or as a result of conservation action (e.g. the Million Ponds Project). Nevertheless, in many regions the number of ponds in the modern landscape is still likely to be the lowest in recorded history, with 80% of remaining ponds in the UK existing in a degraded state (Williams et al. 2010) consistent with other wetland habitats (Defra 2011).

The loss of ponds can threaten the persistence of metacommunities when distances between extant ponds begin to exceed the dispersal abilities of the species they support. Species populations that become isolated by pond loss are at a greater risk of local extinction when faced with environmental disturbances or pollution since they lack nearby habitats from which to source recolonists (Tischendorf and Fahrig 2000; Petersen and Masters 2004; Caquet et al. 2007)⁠. Within the network, connectivity to large ponds is important as these often support source populations (Van Geest et al. 2003; Sondergaard et al. 2005; Hill et al. 2015) consistent with source-sink island biogeography (MacArthur and Wilson 1967). Equally, connectivity to small ponds is also important since these are more likely to be fishless and serve as important reservoirs of aquatic invertebrates, amphibians and macrophytes (Oertli et al. 2002; Sondergaard et al. 2005; Scheffer et al. 2006). The overall spatial configuration and topology of the pond network (locations and distances between habitats) is thus a key consideration for freshwater biodiversity conservation (Biggs et al. 1994; Boothby 1997; Lundkvist et al. 2002; Jeffries 2005) and questions remain as to how the loss of ponds affects the metacommunity structure of the wider network. Through spatial analyses such as graph theory (Harary 1969), it is possible to determine the extent to which pond loss has fragmented the pond network, threatening species metapopulations.

Graph theory has recently emerged as a powerful tool to evaluate the connectivity of habitat networks and the movements of wildlife and genes (Garroway et al. 2008), and here we apply it to investigate the possible impacts of urbanisation on pond networks. In graph theory, networks are distilled into graphical form with nodes representing habitat patches, and edges indicating the existence of functioning connections or ‘ecological flux’ between node populations (Urban et al. 2009). Traditional applications of graph theory in the field of ecology have focused on modelling species networks, such as food webs, plant-pollinator mutualistic relationships or host-parasitoid webs (e.g. Proulx et al. 2005; Bascompte et al. 2006). To date, graph theory approaches have focussed on terrestrial habitat networks (e.g. Laita et al. 2010; Gurrutxaga et al. 2011; Decout et al. 2012) and application to aquatic systems has been largely confined to riverscapes (Erős et al. 2011; Segurado et al. 2013; Eros and Campbell Grant 2015), with scant application to lentic systems (Ishiyama et al. 2014).

Percolation theory, the science of clustering or clumping in random networks (Stauffer 1987), can be used to complement graph theoretical analyses in order to identify important network characteristics. Percolation analyses can elucidate network redundancy or robustness where, for example, apparently redundant nodes provide alternative dispersal pathways should any nodes be lost or impacted (Laita et al. 2011). Transposed into analyses of landscape connectivity, percolation theory is the quantitative analysis of connectivity in spatially structured systems (With 2002). Frequently, percolation analyses are undertaken to reflect known dispersal ability of a focal organism or organisms in order to gain an understanding of the relative connectedness of the network (O’Brien et al. 2006; Reunanen et al. 2012; Ishiyama et al. 2014).

Together, graph and percolation theory can be used to gain a strategic oversight of a habitat network (Galpern et al. 2011) and help identify areas of the network with high ecological flux for management planning or policy formation (Fall et al. 2007; Stewart-Koster et al. 2015). For urban areas this could yield better outcomes for nature conservation effort where resources may be limited. Without consideration of the spatial configuration of habitat there remains a risk that, notwithstanding the potential but largely unknown influence of garden ponds, as urban development continues pond networks could become increasingly fragmented and less resilient to multiple environmental stressors and climate change.

Ponds are a good candidate for graph theory analysis (Moilanen 2011), being discrete habitats linked by dispersal of aquatic biota (e.g. invertebrates), many of which live exclusively within pond networks (Céréghino et al. 2007). In this study, major changes in the structure and connectivity of a pond network within Birmingham, a heavily urbanised region of the UK, were identified for a 105 year period (*ca*1904 – 2009). The structure of the pond network was assessed over time by digitising ponds present on historical Ordnance Survey mapping and the resilience of the network determined by analysing changes in pond distribution, area and number in relation to shifts in land-use. We tested three hypotheses: 1) that considerable pond loss would be observed over the 105 year sequence, 2) pond losses would be strongly associated with urbanisation, and 3) the structural robustness of the Birmingham pond network would decline as the number of ponds in the network decreased.

## Materials and methods

### Study area

**Fig. 1:**
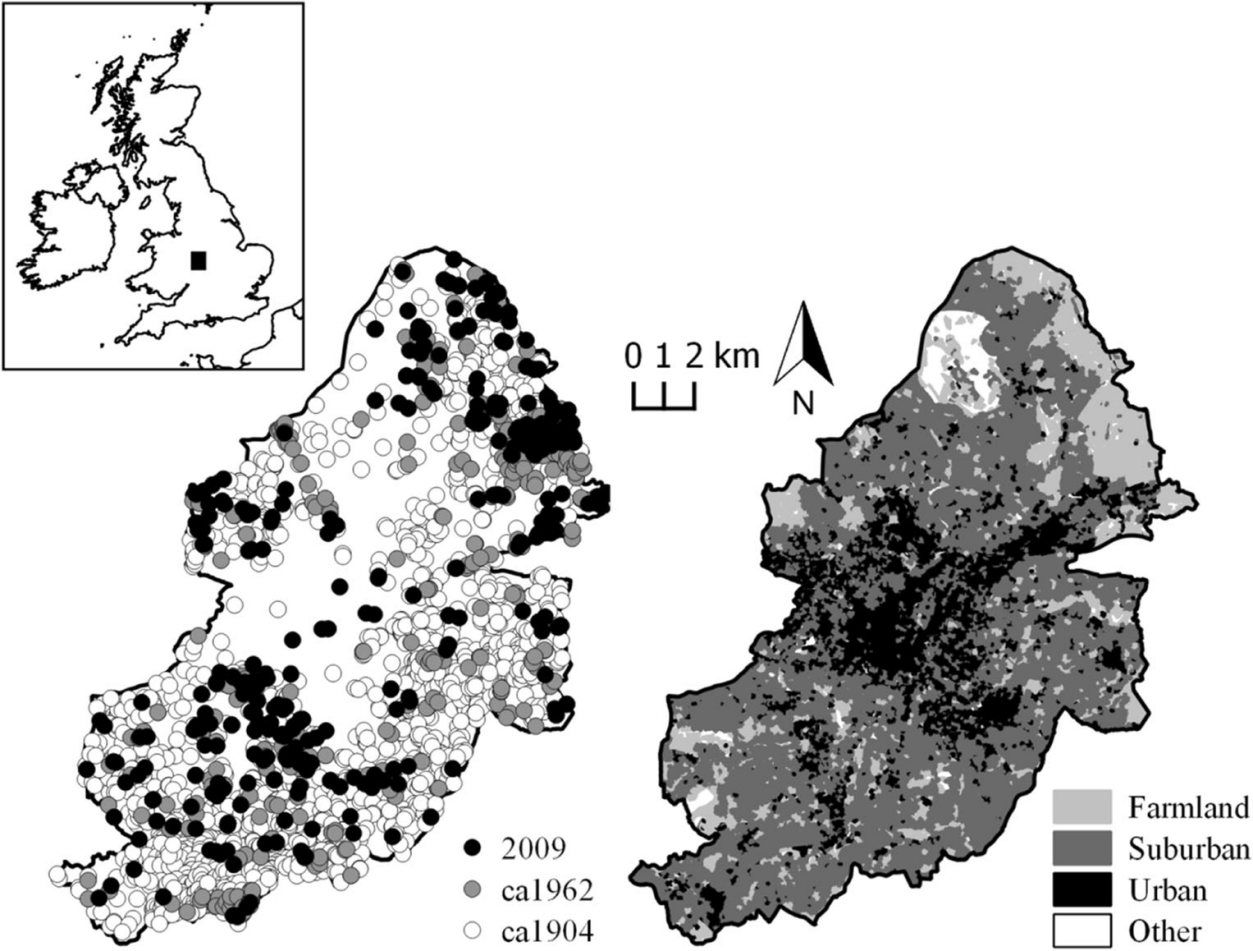
The Birmingham city administration area (study extent) showing historical pond locations, indicative land-use (Land Cover Map 2007) and United Kingdom context (inset)

Birmingham (268 km^2^, 1.1M inhabitants) is located within the Midlands region of the UK (Fig. 1). The area has a rich industrial history of mining and manufacturing, however land-use within contemporary Birmingham comprises mostly of built-up areas and gardens (75% cover), improved grassland, including public parks and gardens (12%), arable and horticulture (8%), mixed, broadleaved or coniferous woodland (4%) and other habitats (1%) (data derived from Land Cover Map 2007). Water bodies (<1% by area) found in the area include rivers, streams and canals as well as lakes, reservoirs and ponds. Ponds with surface area up to 2ha (Biggs et al. 1998), the focus here, are widely dispersed across the region and range from small garden ponds, storm water basins, shallow, naturalised wetlands and ex- marl pits to concrete-lined ornamental ponds within parks.

### Pond digitisation

Data for historical analyses of the pond network in the Birmingham administrative area were derived from three historical map layers, accessed and downloaded in individual 1km^2^ raster tiles from EDINA Digimap and processed in ArcGIS 9.3 (ESRI, Redlands, CA, USA). First, the County Series 1^st^ revision maps of Birmingham (Edina Historic Digimap Service 2012a) were published 1903-1905 (hereafter *ca*1904). Second, the National Grid overhaul and re-survey (Edition A) (Edina Historic Digimap Service 2012b) were published 1943-1995 (hereafter as *ca*1962). Both *ca*1904 and *ca*1962 were mapped at 1:2500 scale which enabled landscape features with an area of 16m^2^ or larger to be identified, although smaller, isolated or significant features may also be mapped (Oliver 2005). The third and most contemporary dataset (2009) was derived from Ordnance Survey MasterMap (EDINA Digimap Ordnance Survey Service 2009). A drawback of each of these mapping methods is that the number of temporary and garden ponds are likely to be underestimated (Jeffries 2012).

Ponds were drawn digitally as individual polygons using ESRI’s ArcScan tool pack extension which permitted surface area calculation. Where possible, curvilinear features without OS map annotation were cross-checked against the other data layers to elucidate pond presence (Thornhill 2013).

### Land-use

Land use surrounding each pond was determined for each time period by fitting to Land Cover Map 2007 (LCM2007; Morton et al. 2011) Broad Habitat classifications and grouped accordingly; Farmland (arable and horticulture), Grassland (improved grassland, neutral grassland), Open/scrub (scrub), Suburban and Urban (differentiated by the continuity of urban component; Fuller et al. 2002), Woodland (broadleaved and coniferous woodland). For *ca*1904 and *ca*1962 this was achieved by examining map annotations and symbols, and for 2009 by extracting the land-use for each pond directly from LCM2007 coverage data. Using the same method, subsequent land-use was also recorded where ponds were lost (i.e. not drawn) or created over time.

### Graph analyses (minimum spanning trees)

**Fig. 2:**
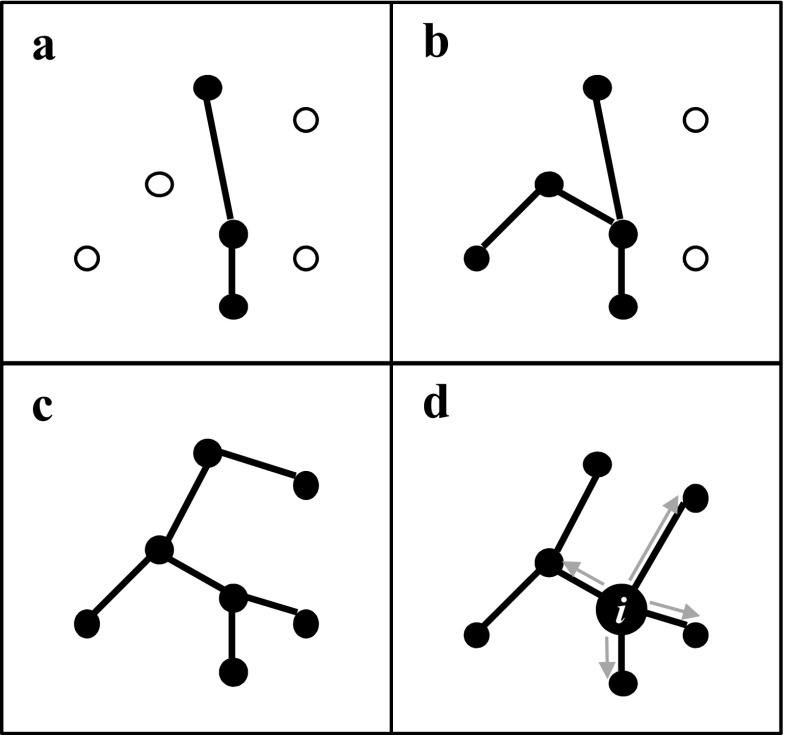
Hypothetical graph arrangements within a pond network. Solid circles indicate connected nodes (ponds) and lines indicate edges (functional connections) where (**a**) is a path, (**b**) a tree, (**c**) a minimum spanning tree (MST) and **d**) a flux-weighted MST which deviates from the MST due to the increased surface area of node i, grey arrows indicate the direction of net ecological flux

Within a graph, edges are considered binary where nodes are connected or not, or they can be quantitative based on probability of connection relative to the distance between them (Dale and Fortin 2010). A path is an unbranched route across a graph in which no node is revisited (Fig. 2a). Multiple connected paths, provided that no closed circuits are created, result in a tree (Fig. 2b), and a tree that connects all nodes within the graph is called a spanning tree. The minimum spanning tree (MST) is the tree that accumulates the least cost (e.g. distance) in connecting all nodes within a graph (Fig. 2c). Urban and Keitt (2001) recommended that conservation efforts should concentrate on the MST as it allows for dispersal across the entire network. Whilst more complex aspects of graph theory can be applied, the MST is likely to indicate the key corridors for the movement and exchange of organisms, or 'backbone' of connectivity (Bunn et al. 2000; Urban and Keitt 2001; Fall et al. 2007) and can be weighted by edge or node features such as surface area. In any graph, metrics such as the betweenness centrality (BC_k_, Freeman 1977) can also be calculated in order to identify the likely importance of nodes as hubs of connectivity or stepping stones (Minor and Urban 2007) for the wider network. For a full review of definitions and terms see (Urban et al. 2009; Kivelä et al. 2014).

MST metrics were calculated based on distance and pond surface area for each historical pond network as determined by Urban and Keitt (2001). First, a Euclidean distance matrix is calculate for all ponds within the network. A probability matrix is then constructed by applying a negative-exponential decay function, within which the steepness of the distance to probability of dispersal relationship is determined by a distance decay conversion factor (Loro et al. 2015). Finally in this factor, a reference distance is used that has ecological relevance (Ribeiro et al. 2011). Here, a distance of 1km was selected, broadly consistent with knowledge of aquatic organism dispersal including insects (e.g. Conrad et al. 1999; Angelibert and Giani 2003) and amphibians such as the newt *Triturus cristatus* (Boothby 1999; Kupfer and Kneitz 2000).

Calculation of a flux-weighted MST_f_ is achieved with the inclusion of relative patch size within the network where larger nodes contribute a higher degree of flux based on the assumption that they contribute larger and more stable populations (Erős et al. 2011; Segurado et al. 2013; Fig. 2d). Minimum spanning trees were derived using the package 'igraph' (Csardi and Nepusz 2006) for R Statistical Software (R Core Team 2017). The igraph package also allowed the computation of a number of diagnostics including average edge length, number of sub components (or clusters) and betweeness centrality (BC_k_) (Freeman 1977). The value of BC_k_ for a given node is the number of shortest pathways between all pairs of nodes in a graph that require it as a stepping stone, which can also be expressed as a proportion to allow for comparison under different scenarios where there may be differing numbers of nodes (e.g. different historical pond networks).

### Percolation analysis

Percolation analyses were carried out in order to consider connectivity across the pond network with respect to observed dispersal abilities of a range of pond dwelling organisms (see Supplementary material T1). Within percolation analysis, the degree of connectivity in a habitat network can be assessed by deriving an order parameter (e.g. the relative size of greatest connected component (GCC) to the whole network) which defines the transition from a connected system to a disconnected one (Kivelä et al. 2014). The distance at which the GCC is no longer evident is called the percolation threshold and represents a critical fraction of links that needs to be removed to break the network into disconnected components (Kivelä et al. 2014). In a pond network with a greater degree of robustness where redundant nodes are abundant, the percolation threshold will occur at a lower distance as the GCC is preserved for longer. The proximity of the percolation threshold to known dispersal abilities can therefore reveal information about the relative accessibility of the network to a given organism. Percolation analyses were carried out using EDENetworks (Kivelä et al. 2014).

## Results

### Pond habitat loss and turnover through time

Between *ca*1904 and 2009 there was an 82% decline in the number of ponds across Birmingham (Table 1), reflecting a net loss of 1573 ponds. Most (73%) ponds were lost between *ca*1904 and *ca*1962 reflecting an average loss of 15 ponds per year. Pond density declined from 7.1 km^-2^ in *ca*1904 to 2.0 km^-2^ by *ca*1962 and to 1.3 km^-2^ in 2009. Over time, the mean surface area of ponds increased (by 204%) as many small waterbodies were lost and larger ones retained (Table 2). This shift was statistically significant early in the sequence (1904 and *ca*1962, Mann-Whitney, P < 0.05), but not later (*ca*1962 and 2009, Mann-Whitney, P = 0.12). The total area of all habitat within the Birmingham pond network declined by 46% between *ca*1904 and 2009, although the retention of larger ponds offset the rapid loss of individual sites. As a proportion of the entire Birmingham administrative area, pond coverage was 0.36% in *ca*1904, 0.22% in *ca*1962 and 0.20% in 2009.

**Table 1:** International comparison of pond loss (adapted from Wood et al. 2003; Globevnik and Kirn 2009; Fairchild et al. 2013; Hassall 2014)

Region	Country(s)	Period	Total loss (%)	Annual loss (%)	Land-use	Source
Bedfordshire	England	1910 – 1981	82	1.15	Intensive arable	Beresford and Wade (1982)
**Birmingham**	**England**	**1904** – **2009**	**82**	**0.78**	**Urban**	**This study**
Cambridgeshire	England	1840/90 – 1990	< 68	< 0.68	Intensive arable	Jeffries and Mills (1990)
Cardiff City	Wales	1880/86 – 1997	< 80	< 0.72	Urban	Rich (1998)
Cardiff County	Wales	1951/61 – 1997	< + 54	< + 1.5	Rural	Rich (1998)
Cheshire	England	1870 – 1993	61	0.50	Rural and urban	Boothby and Hull (1997)
Clywd	Wales	1840/90 – 1990	< 32	< 0.32	Arable and pasture	Jeffries and Mills (1990)
Durham	England	1840/90 – 1990	< 41	< 0.41	Arable and pasture	Jeffries and Mills (1990)
Edinburgh	Scotland	1840/90 – 1990	< 6	< 0.06	Urban	Jeffries and Mills (1990)
Essex (selected areas)	England	1870 – 1989	< 69	< 0.58	Mixed	Heath and Whitehead (1992)
Huddersfield	England	1985 – 1997	31	2.60	Urban/ industrial	Wood et al. (2003)
Huntingdonshire	England	1890 – 1980	56	0.68	Mixed	Beresford and Wade (1982)
Leicestershire	England	1840/90 – 1990	< 60	< 0.60	Intensive arable	Jeffries and Mills (1990)
London region	England	1870 – 1984	> 90	0.79	Mixed	Langton (1985)
Midlothian	Scotland	1840/90 – 1990	< 23	< 0.23	Arable and pasture	Jeffries and Mills (1990)
North Leicestershire	England	1934 – 1979	60	1.33	Mostly pasture	Beresford and Wade (1982)
SE Northumberland	England	1846/69 – 2005/2008	< + 15.8	< + 0.12	Mixed	Jeffries (2012)
Sussex	England	1977 – 1996	21	1.10	Pasture (dewponds)	Beebee (1997)
N Rhine Westphalia	Germany	1963 – 1986	> 40	> 1.7	Mixed	Glindt (1993)
South Berlin	Germany	1880 – 1980	81	0.8	Forest	Sukopp (1981)
Barnim	NE Germany	1839 – 1989	33.3	0.13	Agricultural	Schneeweiss and Beckmann (1999)
Wielopolska	Poland	1890 – 1941	56	1.1	Agricultural	Ryszkowshi and Balazy (1995)
Pomerania	Poland	1888 – 1980	< 70.2	< 0.76	Agricultural	Pieńkowski (2003)
Rio Grande do Sul	Brazil	1905 – 2005	90	0.9	Wetlands, rice-fields	Guadagnin et al. (2005)
Chianti Hills	Italy (Tuscany)	1939 – 1999	35	0.58	Vineyards	Scoccianti (1999)
Orbotello lagoon area	Italy (Tuscany)	1939 – 1999	12.5	0.21	Coastal, shrub	Scoccianti (1999)
Trieste	Italy / Slovenia	1979 – 1998	70	3.7	Karstic plateau wetlands	Bressi and Stoch (1998)
Žumberak-Samoborsko Gorje	NW Croatia	1935 – 2005	51.8	0.74	Nature reserve	Hutinec and Struna (2007)
SE Pennsylvania and N Delaware (Brandywine)	United States	1883 – 1936	-	2.70*	Piedmont	Fairchild et al. (2013)
	United States	1946 – 2005	-	0.16*	Piedmont	Fairchild et al. (2013)
Sweden		1914 – 1970	55	1.0	Mixed	Bjureke et al. (1976)
Netherlands		1900 – 1989	90	1.0	Mixed	Weinreich and Musters (1994)
England and Wales		1880 – 1920	57.5	1.41	Mixed	Rackham (1986)
United Kingdom		1990 – 1996	7.4	1.23	Mixed (lowland ponds)	Williams et al. (1998a)
United Kingdom		1900 – 1990	75	0.78	Mixed	Bailey-Watts et al. (2000)
United Kingdom		1998 – 2007	+ 12.5	+ 1.4	Mixed	Williams et al. (2010)

*Based on exponential decay function

**Table 2:** Birmingham pond network general and minimum spanning tree (MST_f_) summary statistics

Measure	*ca*1904	*ca*1962	2009
Number of ponds	1914	524	341
Pond density (km^-2^)	7.14	1.95	1.27
Mean surface area (m^2^ ±1SE)^†^	508 (± 38.6)^a^	1126 (± 120.1)^b^	1537 (± 176.4)^b^
Total habitat area (ha)	97.3	59.0	52.4
Ponds created^*^	-	171	112
Original ponds^**^	-	353	171
MST_f_ mean betweeness (BC_k_ ±1SE)	0.020 (± 0.002)	0.056 (± 0.006)	0.062 (± 0.007)
Average MST_f_ edge length (m)	462	443	687
Percolation threshold (m)	811	1559	2361

^†^Lettering denotes significant differences (Mann-Whitney, P <0.05)

^*^Created since last mapping series and may be subsequently lost

^**^Present throughout 105 year study period

Changes in the total number of ponds masks a considerable amount of turnover within the pond stock over time (Table 3). Half (50.1%, 171 sites) of the ponds reviewed in 2009 were over 105 years old (present at *ca*1904 census) and half (49.9%, 170) were younger, created and not subsequently lost between *ca*1904 and *ca*1962 (17%, 58 sites) or between *ca*1962 and 2009 (32.3%, 112). The 171 ponds that persisted throughout the study represented just 8.9% of the number that were present in *ca*1904. Whilst overall mean surface area of ponds increased, the mean surface area of ponds that were present throughout the study period decreased over time from 2413m^2^ (*ca*1904) to 1935m^2^ (*ca*1962) to 1919m^2^ (2009) which represents an overall 21.5% decrease in average surface area.

**Table 3:** Number of ponds (/km^2^) lost and created within different land-uses across the Birmingham pond network. Percentiles, in parenthesis, reflect total numbers lost or created by the latter mapping sequence

	*ca*1904 to *ca*1962			*ca*1962 to 2009		
Land-use	Lost	Created	Net	Lost	Created	Net
Farmland	0.86 (14.7%)	0.13 (19.9%)	-0.73	0.23 (21.0%)	0.07 (16.1%)	-0.16
Combined*	0.03 (0.6%)	0.00 (0.0%)	-0.03	0.00 (0.0%)	0.00 (0.0%)	0.00
Grassland	0.43 (6.9%)	0.10 (15.2%)	-0.31	0.16 (14.6%)	0.06 (15.2%)	-0.10
Open / scrub	0.28 (4.8%)	0.12 (18.7%)	-0.16	0.01 (0.3%)	0.01 (3.6%)	+0.01
Suburban	3.43 (58.9%)	0.16 (25.7%)	-3.26	0.52 (47.5%)	0.13 (30.4%)	-0.40
Urban	0.50 (8.6%)	0.06 (9.9%)	-0.44	0.10 (9.2%)	0.07 (17.9%)	-0.03
Woodland	0.29 (4.9%)	0.06 (9.4%)	-0.23	0.08 (0.7%)	0.07 (17.0%)	-0.01
Unknown	0.03 (0.6%)	0.01 (1.2%)	-0.03	0.00 (0.0%)	0.00 (0.0%)	0.00
Total	-5.83	+0.64	-5.19	-1.10	+0.42	-0.68

*Combined with or split from another pond or other wetland habitat e.g. river / lake

Many ponds were originally associated with farmland, however, the number of such ponds decreased rapidly from 71% of total pond numbers in *ca*1904 to just 15% by 2009 (Table 4). Much pond loss was attributable to suburban expansion, which was the land-use identified to have replaced lost ponds in 875 (3.26 /km^2^) and 106 (0.40 /km^2^) cases between *ca*1904 and *ca*1962, and *ca*1962 and 2009 respectively (Table 3). Net losses within farmland were the second highest. The number of ponds associated with, or enveloped by, suburban areas and areas of grassland (including public parkland and golf courses) increased by 2009 to account for a total of 57% of those present, from just 8% in *ca*1904. The number of ponds associated with urban / industrial land-use was consistently low, at no point accounting for more than 8.5% (0.11 /km^2^) of ponds present. Though suffering an overall loss in numbers, the number of ponds associated with woodlands relative to all those within the network consistently increased through time from 9.6% (*ca*1904) to 17.6% (2009).

**Table 4:** Land-use associated with pond presence through time within the Birmingham pondscape

Land-use	*ca*1904	*ca*1962	2009
Farmland	5.09 (71.3%)	0.62 (31.9%)	0.19 (14.7%)
Grassland	0.37 (5.1%)	0.34 (17.2%)	0.31 (24.6%)
Open / scrub	0.52 (7.3%)	0.26 (13.2%)	0.02 (1.8%)
Suburban	0.21 (2.9%)	0.28 (14.3%)	0.42 (32.8%)
Urban	0.24 (3.3%)	0.15 (7.8%)	0.11 (8.5%)
Woodland	0.68 (9.6%)	0.30 (15.3%)	0.22 (17.6%)
Unknown	0.04 (0.6%)	0.01 (0.4%)	0.00 (0.0%)
Total ponds present	1914	524	341

In total, 2195 geographically distinct ponds were present at any one time throughout the 105 year study period. A total of 283 were created and 1856 lost (Table 3). The study revealed the origins and use of a subset (334 ponds, 15%) of Birmingham's ponds. Most, often small (mean surface area 493m^2^), were built for purposes of landscaping (101 ponds, 30%) as either ornamental features (67 ponds) or moats (39 ponds). The minerals industry was the second most frequent contributor (81 ponds, 24%), of which just over half were as a result of brickworks (42 ponds, 52%). The third most important use was for recreational purposes, predominately for fishing, accounting for 18% (60 ponds) whilst other industrial uses (e.g. disused reservoirs, sludge lagoons, mill ponds) comprised a further 16%. Ponds created as a result of other industrial uses or for recreational purposes (e.g. fishing) were typically large relative to this audit (mean surface area 4129m^2^). Very few ponds (20, 6%) appeared to be created through natural processes; the majority (84%) of the subset were thus artificial, having been created to support human activities although it was not possible to ascertain the origins of 87% of ponds that were already present in *ca*1904.

### Pond network resilience through time

Changes in the pond network were characterized by a 49% increase in MST_f_ mean edge length from 462m to 687m, meaning that ponds within the 2009 network were considerably more isolated with fewer neighbours. Mean centrality values (BC_k_, normalised for each time period) showed an increase of 210%, suggesting that biodiversity movement across the network was increasingly reliant upon fewer ponds (Table 2).

**Fig. 3:**
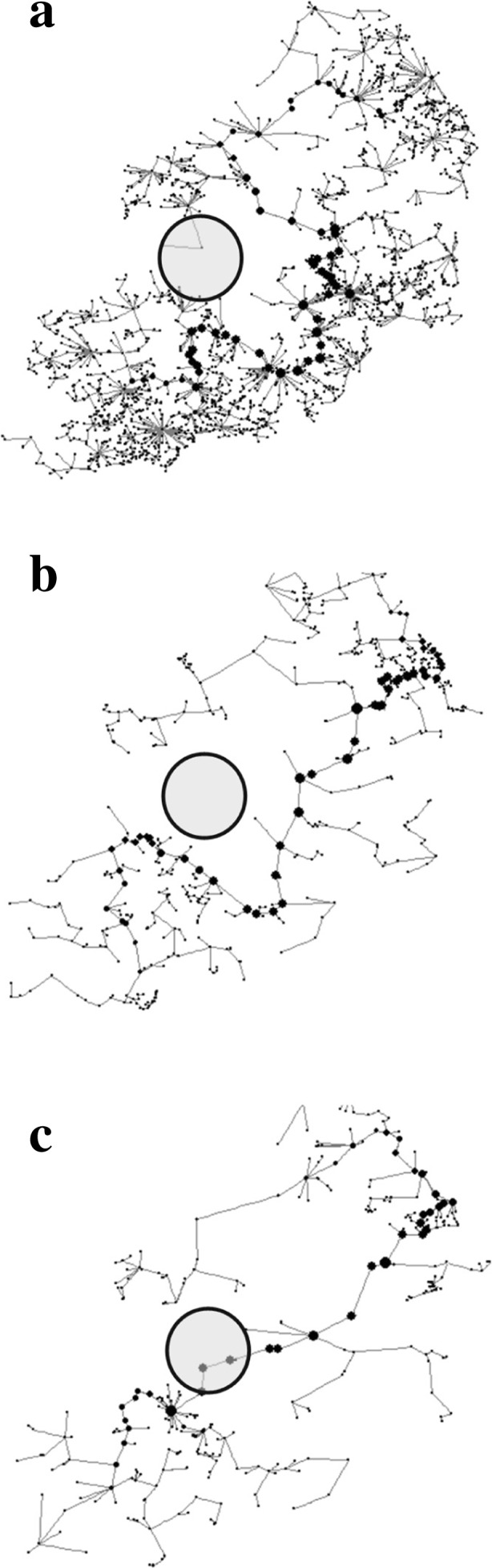
Flux weighted minimum spanning trees for the Birmingham pond network for (**a**) *ca*1904, (**b**) *ca*1962 and (**c**) 2009. Nodes represent ponds, larger nodes indicate higher BC_k_ relative to the network. Lines represent edges. Grey circles represent the location of the contemporary Birmingham city centre

In the *ca*1904 and *ca*1962 landscapes the MST_f_ routed outside of the central area of Birmingham through areas of higher pond density to the south and to the east (Fig. 3b, c). In *ca*1904, three distinct patches of high pond density were apparent in the northeast, east and south of Birmingham centre. However, the numbers of ponds in these areas were much diminished by 2009 (Fig. 3a) where up to 30 ponds km^-2^ were lost between *ca*1904 and *ca*1962. Although much overshadowed by the impact of urban expansion affecting the wider pond network, several new ponds have been created as part of modern developments since *ca*1962 that marginally improve pond density within some central areas of Birmingham. Due to the loss of ponds to the south and east in particular, and a slight gain in the city centre, the 2009 MST_f_ re-routes through the central area (Fig. 3a).

**Fig. 4:**
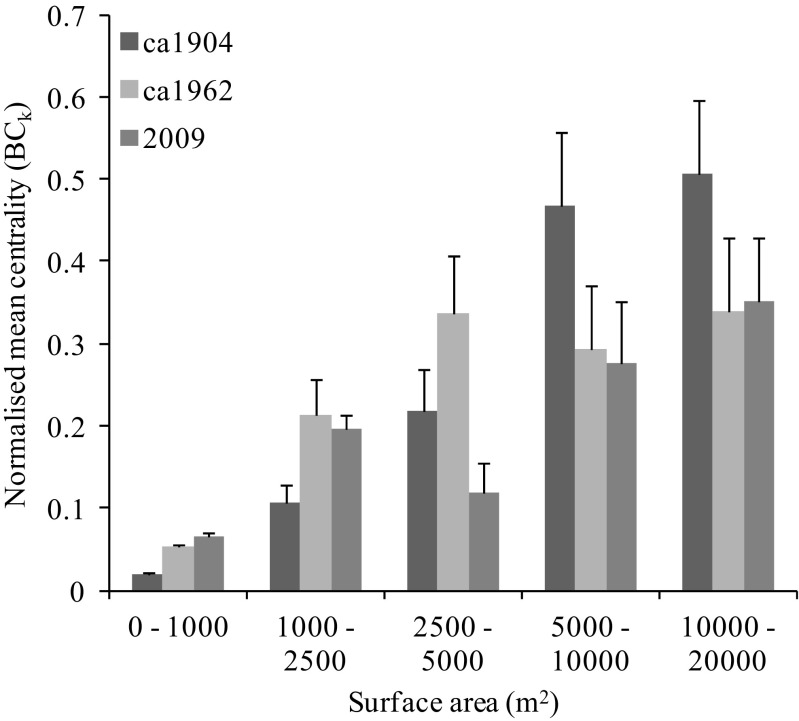
Distribution of betweeness centrality measures between ponds (nodes) of different size classes within the flux-weighted MST_f_ for each historical pond network

Analysis of the distribution of centrality values across different size classes of pond within the MST_f_ demonstrated decreasing levels of redundancy in the pond network through time (Fig. 4). Across the *ca*1904 network the largest ponds (>5000m^2^) are also the most central typically with BC_k_ values of approximately 0.5. Since *ca*1904 however, despite the retention of larger ponds, they are much less central (lower BC_k_ values) indicating an inadequate network of stepping stones to allow the MST_f_ to route through habitats more likely to provide source populations.

**Fig. 5:**
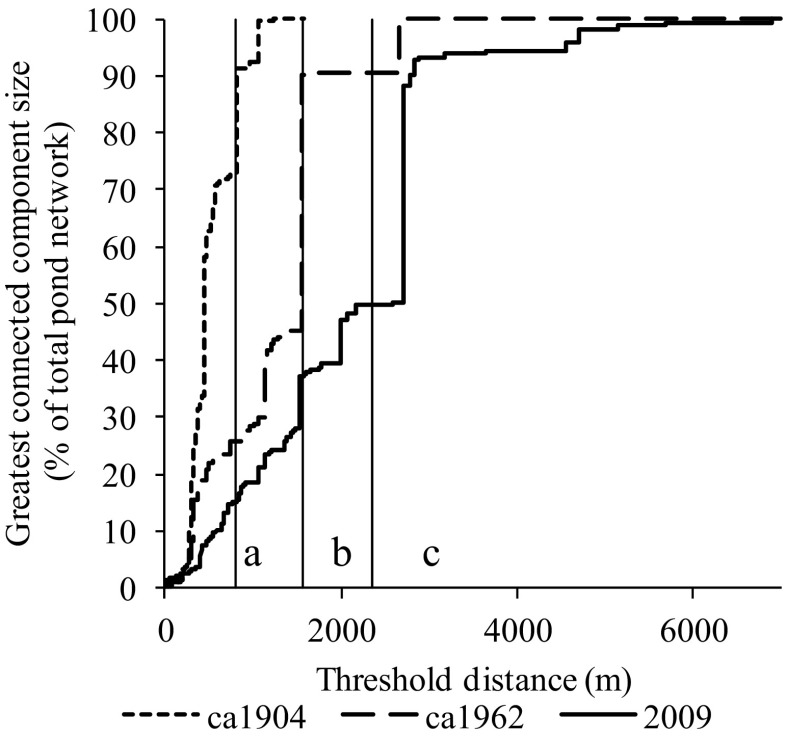
Percolation graphs for Birmingham’s historical pond networks demonstrating the impact of a decreasing threshold distance upon the greatest connected component (GCC) relative to the number of nodes within the pond network. Percolation thresholds also shown as solid vertical lines for (**a**) *ca*1904 - 811m, (**b**) ca1962 – 1559m and (**c**) 2009 – 2361m

**Fig. 6:**
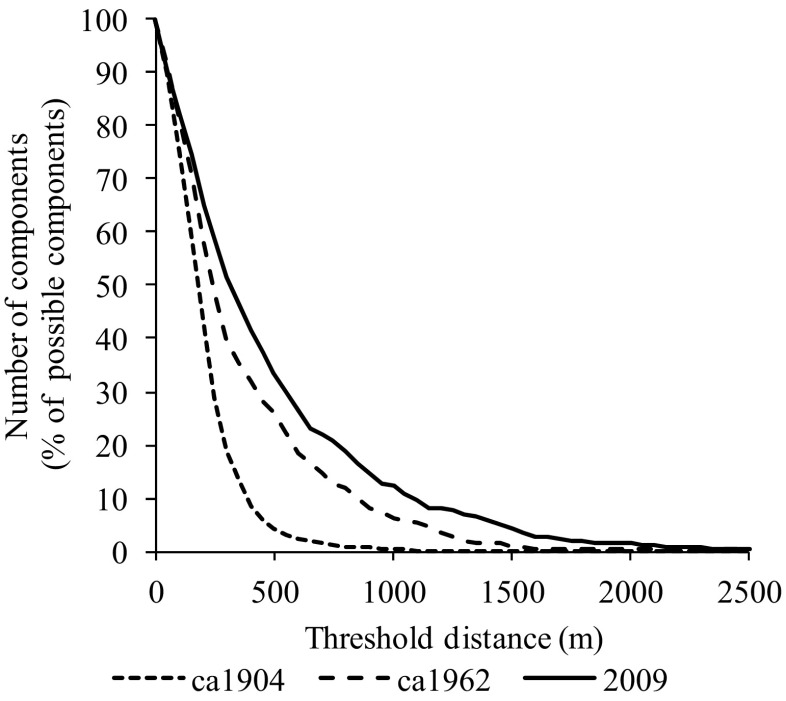
Fragmentation of Birmingham’s historical pond networks demonstrated by the number of sub-components relative to the maximum number possible (i.e.the total number of nodes), generated as a result of a decreasing threshold distance

In the *ca*1904 network, more nodes are retained within Greatest Connected Component (GCC) as the distance threshold is decreased resulting in a percolation threshold of 811m (Fig. 5). However, as ponds are lost over time, the percolation threshold increased to 1559m by *ca*1962 to 2361m by 2009 (Table 2). If a dispersal threshold of 1km was applied to the networks, most (92.4%) of ponds would comprise the GCC in the *ca*1904 pondscape whilst it would only be comprised of 148 (28.2%) and 63 (18.5%) ponds by *ca*1962 and 2009 networks respectively. Similarly, the total number of sub-components created by applying a 1km threshold proportionally increases over time, thus in *ca*1904, 42 sub-components were created from a possible 1914 (0.4%), whilst in *ca*1962 and 2009 33 (6.3%) and 7 (12.3%) sub components were created respectively (Fig. 6).

## Discussion

This study aimed to assess how Birmingham's network of pond habitats has altered in response to increasing urbanisation over a 105 year period and consider changes in network robustness and implications for biodiversity. A rate of pond loss in Birmingham between *ca*1904 and 2009 of 0.78% per annum is comparable to that of London (0.79%) between 1870 and 1984 (Table 1), and an 82% total loss in total pond numbers between *ca*1904 and 2009 ranks Birmingham second highest in the UK (accept hypothesis one), behind urban London and comparable to losses in Bedfordshire's intensively agricultural landscape (Beresford and Wade 1982). Whilst there are few records of ponds loss outside of the UK, losses in Birmingham appear greater than nationwide losses observed in Sweden (Bjureke et al. 1976) and comparable to those in the Netherlands (Weinreich and Musters 1994). Since *ca*1962, the rate of annual pond loss has declined to 0.1%, consistent with the nationwide disappearance of ponds reported by Biggs et al. (2005). Therefore, pond-dwelling organisms within Birmingham are likely to rely upon fewer ponds, potentially rendering their metacommunities less resilient to stochastic events such as pollution or deterministic changes such as global climate change. Ponds that have remained throughout the study (171 ponds) have a notably reduced surface area, which may suggest the occurrence of natural successional processes e.g. vegetation encroachment, or development pressures.

### Pond resource turnover, loss and creation

As reported by a number of other authors (Williams et al. 1998a; Jeffries 2012), the raw numbers mask a high turnover in pond resource. The turnover in stock is clearly linked to land-use change and strongly driven by the process of urbanisation (accept hypothesis two) not dissimilar to the impact of coastal urbanisation upon an estuarine wetland network (Dou and Cui 2014). Here however, former farmland field ponds were either lost to, or enveloped by, suburban development as others (few by comparison) were built as part of those developments. These findings accord with several studies of Birmingham's demography and changing landscape, where the population of Birmingham has increased from approximately 500,000 in 1900 to 1M by the early 2000s (Haynes 2008; University of Portsmouth 2017), which coincided with the expansion of Birmingham city centre throughout the 20th century as villages and hamlets coalesced into suburbs through industrial and residential development on former agricultural land (Axinte 2015).

The vast majority of ponds lost within Birmingham were probably artificial in nature, however in a highly altered landscape they are likely to act as surrogates for natural habitats and studies have shown artificial ponds to have high conservation value (Vermonden et al. 2009; Hill et al. 2017; Thornhill et al. 2017). Nevertheless, it is apparent that many ponds are more isolated from their neighbouring habitats and there is a large body of evidence in the published literature that this degeneration of the pond network with less connected nodes has large implications for local and regional biodiversity (e.g. Table 1).

A recent reduction in the rate of pond loss within Birmingham may be due to the retention of larger ponds, which are frequently located in public green spaces or used for recreation (e.g. boating and fishing). Such cultural landmarks may receive protection through local authority planning policies or legislation. Applying conventional island biogeography (MacArthur and Wilson 1967), the retention of high quality larger ponds could help to preserve some of the network's source populations and reduce the overall impact of pond loss. However, a number of studies have also shown that a cluster of small ponds are key contributors to regional invertebrate biodiversity (Wood et al. 2001; Scheffer et al. 2006; Boix et al. 2012). Reasons for this are complex, however it may be due to an increased fish and waterfowl presence in larger ponds resulting in the exclusion of some invertebrate species and reduction in vegetation complexity (Oertli et al. 2002; Sondergaard et al. 2005; Schilling et al. 2009) or to an increase in the number of habitat niches available across several small ponds (Williams et al. 2004). Nevertheless, the potential ecological value in retaining larger ponds for biodiversity may be compromised in Birmingham due to the loss of smaller stepping stone habitats that would otherwise connect larger ponds to the network by facilitating species dispersal.

### Possible implications for the biota

As a result of pond loss, the 2009 percolation threshold (2.36km) suggests that for many aquatic biota the Birmingham pond network is comprised of a series of sub-components, the number of which can be inferred through statistical thresholding (Fig. 6). A percolation threshold of 811m and average MST_f_ edge length of 462m indicated that more frequent exchange of biota between ponds was likely in the *ca*1904 pond network, which would have historically allowed more rapid recovery of local populations from stochastic events. For invertebrates, the exception may be a small percentile of species populations which make long distance movements (Conrad et al. 1999) which may be sufficient to maintain genetic diversity (Lowe and Allendorf 2010) but not population recovery.

The majority of macroinvertebrate dispersal studies have focused on Odonates, though nearly all suggest that dispersal, particularly of Zygopterans, beyond 1km is rare and due to high levels of philopatry the majority of movements are constrained to less than 100m (Rouquette and Thompson 2007; Supplementary Material Table T1). Though potentially less severe due to stronger dispersal tendencies, the scenario is likely to be similar for some Hemipterans (Briers 1998) and Diptera (Service 1997). Less clear is the impact that pond loss is likely to have had on non-winged (i.e. passive) invertebrate dispersal (e.g. Gastropods and leeches (Hirudinea)) which are largely incapable of self-dispersal between habitats and rely on vectors (Bilton et al. 2001).

Analysis of the spatial configuration of pond networks alone suggests that the majority of the 2009 Birmingham pond network has too few ponds that are typically too far apart to sustain populations of the European protected amphibian, *Triturus cristatus*. *T. cristatus* is generally considered to disperse up to 250m (Langton et al. 2001), with few studies reporting movements up to 1km (Kupfer and Kneitz 2000) and optimum pond density for a *T. cristatus* metapopulation is considered to be 4km^-2^ (Oldham et al. 2000), which is seldom achieved by 2009 in this study (mean pond density 1.3km^-2^). These findings may substantiate the suggestion that *T. cristatus* populations across Birmingham are generally thought to have experienced a decline, though limited data exist (The Wildlife Trust 2000).

Although the overall robustness of the Birmingham pond network has clearly declined with probable implications for many pond-dwelling organisms, further study is required to understand the relative significance of impacts to biota with different dispersal modes and strengths (partially accept hypothesis 3). In addition, this analysis does not represent probable losses in temporary ponds (Jeffries 2012) and or the occurrence small garden ponds which are estimated to be present within 10% of UK gardens (Davies et al. 2009). However, whilst valuable as temporary refuges the biodiversity supported by garden ponds has been shown to be a nested subset of field ponds (Hill and Wood 2014) and potentially unlikely to offset their loss.

### Future directions

The present study provides the first application of graph theory to a pond network. However, research in three areas would improve the ecological grounding of such spatial models. First, dispersal across the urban landscape is highly unlikely to be uniform as it is comprised of many obstacles such as roads (Parris 2006) and artificial lighting (Bilton et al. 2001; Smith et al. 2009). Small aquatic invertebrates in particular are difficult to track and efforts have focused primarily on rare species (e.g. Purse et al. 2003; Hassall and Thompson 2012). Second, within habitat quality could not be assessed, yet national studies have identified a decline in the quality of ponds (Williams et al. 2010), which could suggest that many included here may not be suitable for colonisation for pollution sensitive taxa. To this end, public participation in data generation (i.e. citizen science; Thornhill et al. 2016) and improvements in remote-sensing (Palmer et al. 2015) are promising avenues. Thirdly, we used a 1km threshold as a broad average of the dispersal ability of the community, however, a more in depth analysis could apply shorter and longer thresholds to better represent the varied abilities of the ecological community to disperse (Galpern et al. 2011), whilst being careful not carry out analysis without sufficient evidence base (Moilanen 2011); thus referring back to point one above.

## Conclusion

The identification of the backbone of an urban pond network by using graph theory and the concept of minimum spanning trees (MSTs) is the beginning of a landscape-scale strategy for the conservation of pond fauna and flora rather than traditional single site management. An extended analysis should be carried out to include the wider pond network such that study boundaries are reflective of natural boundaries. However, this study highlights important clusters and pathways within the current pond stock as well as evidence of a need to improve the networks spatial resilience.

This study finds that ponds have become increasingly scarce in the urban landscape over more than 100 years as they are lost to the process of urbanisation. The loss of ponds since *ca*1904 is considerable, but the rate of pond loss has slowed in recent times. This may be reflective of the types of ponds which are being retained as they are often in the public eye and form part of amenity parkland.

The manner in which this study has been carried out is stepwise and intuitive and can be undertaken with widely available software such that it may be readily repeated across regions and adapted for other landscapes. Landscape managers should ensure that the ponds that remain are of good quality and could use the analytical approach presented here to strategically create ponds in order to reduce the vulnerability of the pond network to further habitat loss. However, opportunities remain to further refine the approach by incorporating inter-habitat resistance to dispersal due to unfavourable land-use.

## Electronic supplementary material

ESM 1 (DOCX 19 kb)
