# Supplementary material for: The application of graph theory and percolation analysis for assessing change in the spatial configuration of pond networks
Source: Urban Ecosyst. 2017 Dec 18;21(2):213–25. doi: 10.1007/s11252-017-0724-8 (PMC6560936; doi:10.1007/s11252-017-0724-8)
Supplement: Supplementary file 1 — (DOCX 19 kb) [file 11252_2017_724_MOESM1_ESM.docx]

**Degradation of a pond network during a century of urbanisation: implications for freshwater biodiversity**

Ian Thornhill^1*^, Lesley Batty^1^, Matthew Hewitt^1,^Nikolai R. Friberg^2,3^, Mark E. Ledger^1^

^1^School of Geography, Earth and Environmental Sciences, University of Birmingham, Edgbaston, Birmingham, West Midlands, B15 2TT, United Kingdom

^2^Norwegian Institute for Water Research (NIVA), Gaustadalléen 21, 0349 Oslo, Norway

^3^water@leeds, School of Geography, University of Leeds, Leeds LS2 9JT, United Kingdom

*Corresponding author - ian.thornhill@live.co.uk, +044 (0) 1865 318263

**Supplementary material**

Table T1 Known dispersal abilities of some aquatic invertebrates known to inhabit pond environments from multiple studies across temperate regions

| Species | Order | Family | Dispersal distances | Method | Water body type and landscape | Source |
| --- | --- | --- | --- | --- | --- | --- |
| *C. puella*  *C. pulchellum*  *E. cyathigerum*  *I. elegans*  *P. nymphula*  *L. sponsa* | Odonata | Coenagrionidae  Lestidae | <1% >*ca* 600m  (all species) | MRR | Marl pits;  arable and livestock;  U.K. | Conrad et al., 1999 |
| *S. sanguineum* |  | Libellulidae | Max. 1200m |  |  |  |
| *C. puella*  *C. scitulum* | Odonata | Coenagrionidae | 0.9% >725m  1.5% >725m | MRR | Ponds; Arid, karstic; France | Angelibert & Giani, 2003 |
| *C. mercuriale* | Odonata | Coenagrionidae | 65.7% <50m  1.3% >500m  0.1% >1000m | MRR | Water meadow carriers & ditches;  Meadow, agriculture, urban;  U.K. | Rouquette & Thompson, 2007 |
| *C. mercuriale* | Odonata | Coenagrionidae | <500m  ≤4500m | MRR  DNA | Streams, ditches, agricultural, Switzerland | Keller et al., 2010 |
| *I. pumilio* | Odonata | Coenagrionidae | 67.9% <50m /  87.6% <50m  Max. (♀) 575m /263m | MRR | Springs & flushes;  New Forest / Red River valley (rural-residential & agricultural); U.K. | Allen & Thompson, 2010 |
| *E. cyathigerum* | Odonata | Coenagrionidae | 27% >100m | MRR | Not stated | Garrison, 1978 |
| *N. maculata*  *N. obliqua* | Hemiptera | Notonectidae | Max. >1600m | Exhaustive sweep-netting, seasonally | Dewponds;  Peak District National Park, U.K. | Briers, 1998 |
| - | Diptera | Culicidae | Max. <5000m  Max. <1000m | Unknown | Unknown | Service, 1997 |

MRR – Mark-release-recapture NR – Not reported Max. – Study maximum

*Distance travelled by 50% of collected animals (median)
